# Prevalence and short-term clinical impacts of new-onset diabetes mellitus among patients with COVID-19 in jazan region, Saudi Arabia

**DOI:** 10.1186/s12902-024-01724-z

**Published:** 2024-09-20

**Authors:** Amal H. Mohamed, Majid Darraj, Abuobaida Yassin, Mohammed Somaili, Ahmed Sayed, Omar Oraibi, Mostafa Mohrag, Mohammed Ali Madkhali, Sameer Alqassimi, Mohammed A. Madkhali

**Affiliations:** Internal Medicine Department, Faculty of Medicine, Jazan University, Jazan, Saudi Arabia

**Keywords:** New-onset DM, COVID-19, Jazan region

## Abstract

**Background:**

Diabetes Mellitus is a major predictor for severity and mortality that is increased by 50% in COVID-19 infection. The aim of this study is to estimate the prevalence of new-onset DM among patients with COVID-19 and examined the short clinical outcomes of the disease.

**Method:**

This is a retrospective study of revising electronic medical records to assess the prevalence of new-onset DM in COVID-19 patients and its impact on the severity of the disease. Adult patients with confirmed COVID-19 during the period from June 2020 to December 2021 were enrolled.

**Results:**

725 patients were included. 53.8% of them were males and 46.2 were females, the mean age was 43.35 ± 16.76. 13.2% were diabetics; 2.2% with preexisting DM and 11.0% had new-onset DM. 6.34% had coexisting medical conditions. DKA at presentation was observed in 6 patients (0.8%) of newly diagnosed DM. There is a significant correlation between age and family history (FH), and BMI and new-onset DM (*P* < 0.05). The overall mortality rate was 2.2%, and it was significantly higher in diabetics in comparison to non-diabetics (*P* < 0.001). 8.6% had persistent hyperglycemia after 4 months of follow-up.

**Conclusion:**

The prevalence of COVID-19 related new-onset DM was correlated significantly with disease severity and mortality rate. Age, FH, and BMI, were the major predictors. We recommend that frequent monitoring of blood glucose for patients with COVID-19 infections to detect DM, therefore, prompt treatment can be initiated.

## Introduction

The pandemic of Coronavirus Disease-2019 (COVID-19), has been growing rapidly worldwide since the first report of the infection in Wuhan, China, in December 2019. It has a negative impact on health and psychosocial aspects and even on the economy [1].

There is a back-and-forth relation between diabetes mellitus (DM) and COVID-19. DM, on the one side, is linked to a higher incidence of severe COVID-19, and so optimum glycemic control is essential to reduce the risk of COVID-19 and its severity [2].

Several epidemiological studies in severe acute respiratiry syndrome coronavirus 2 (SARS-CoV-2)-affected areas, as well as data from different health facilities, demonstrated that the severity and rate of death associated with COVID-19 is increased by 50% in diabetic patients than in those without diabetes. This is can be explained by the impaired innate immunity, altering phagocytosis, neutrophil chemotaxis, and cell-mediated immunity which associated with diabetes mellitus [3]. Even in those without diabetes, a minor increase in blood glucose is frequent in COVID-19 patients and is correlated with a bad prognosis [4].

The increased complications and severity risk of diabetes in COVID-19 could still be a reflection of the greater incidence of type 2 diabetes among older individuals. Additionally, diabetes in the elderly has been related to coronary artery disease and other cardiovascular problems, which can explain the correlation between DM and severe COVID-19 infection [3]. 

In patients with COVID-19, however, new-onset DM and severe metabolic consequences of current diabetes, such as diabetic ketoacidosis (DKA) and hyperosmolarity, necessitating extremely high insulin doses, have been reported. These diabetic symptoms are difficult to control and reflect a complicated etiology of COVID-19–related diabetes (CRD) [5]. New-onset CRD is defined as hyperglycemia, in a patient with established COVID-19, an absence of diabetes history with normal HbA1c [6].

The exact mechanisms for new-onset diabetes in individuals with COVID-19 are not clearly understood, but a variety of interconnected mechanisms are likely to engage, including undiagnosed DM, stress hyperglycemia, steroid-induced hyperglycemia, and impacts of SARS-CoV-2 on the beta cell directly or indirectly [4].

In 2003, during SARS coronavirus (SARS-CoV) pandemic, several research investigations which looked into the causes of pancreatic lesions and glucose intolerance in SARS patients, demonstrated that the presence of angiotensin-converting enzyme 2 (ACE2) in the exocrine and endocrine tissues of the pancreas indicated that SARS-CoV could destroy islets and lead to acute insulin-dependent diabetes mellitus [5–7]. SARS-CoV-2 attaches to ACE2 receptors, which are found in metabolic organs and tissues such as pancreatic beta cells, adipose tissue, the small intestine, and the renal tissues and use it to enter inside cells, causing modulatory changes in glucose homeostasis, which could exacerbate the pathophysiology of pre-existing diabetes or result in new disease processes [5]. Other coronaviruses that bind to ACE2 receptors have also been implicated in the development of ketosis, and individuals with SARS coronavirus1 pneumonia have been found to have higher rates of fasting hyperglycemia and new-onset severe DM than those with non-SARS pneumonia [8]. Insulin deficiency plays a crucial role in the effect of SARS-CoV-2 on β cells, through ACE2, and can lead to both type1 and type 2 DM [8, 9].

A number of studies have currently explained how insulin deficiency in type 2 diabetes can be the primary abnormality or that it can be as a result of glucotoxicity to cells following duration of high blood sugar levels brought on by insulin resistance. In type 1 diabetes, insulin deficiency is caused, at least in part, by autoimmune processes [9].

Activation of pro-inflammatory cytokine like interlukein-6 and C-reactive protein, as acute phase reactant, fostering autoimmune response in individual with genetic suspetabilty, as well as alteration in health habits throughout the pandemic are other contributors to the development of CRD [9]. These concerns were addressed by the CoviDIAB Project, which was established to determine the scope and features of new-onset diabetes associated with COVID-19 -related, as well as the pathophysiology, treatment, and consequences of the disease [10].

Thus, these findings support the concept that COVID-19 may have a diabetogenic impact in addition to the well-known stress response related to acute sickness [3]. However, it’s unknown if the changes in glucose homeostasis that emerge with a rapid onset in severe COVID-19 remain or disappear once the infection is eradicated [5].

According to the American Diabetes Association (ADA), new-onset hyperglycemia is diagnosed with fasting blood glucose (FBG) of 5.6–6.9 mmol/l and or HbA1c 5.7-6.4% in the absence of previous hyperglycemia, while the diagnosis of new-onset diabetes required two abnormal results of FBG of 7.0 mmol/l or HbAIc of < 6.5%, or random blood sugar of 11.1mmol/l in addition to features of hyperglycemia, with no history of diabetes mellitus previously [11]. CRD is a clustering sign of multi-organ damage that predicts poor results and mortality. It will be beneficial to have an awareness of how COVID-19-related diabetes developed the disease’s natural history, and its proper therapy [12]. Commonly insulin is used efficiently in the management of new-onset diabetes. Patients with type 1 DM required close monitoring of blood glucose and ketones in urine, and insulin doses should be adjusted. Oral hypoglycemic drugs which can lead to volume depletion should be avoided. Reduction of doses of these agents with adjustment is recommended [3].

The research on COVID-19–related diabetes could possibly lead to the discovery of new disease pathways and management [8]. However, a significant association and impact of previously diagnosed DM on the clinical course and outcome of COVID-19 was considered in many recently published studies, only a limited data is available on the new-onset of DM in COVID-19 patients and its impact on clinical progression, and its prognosis following COVID-19 recovery.

The main objective of the study is to estimate the prevalence of new-onset diabetes in COVID-19 patients in the Jazan region and to highlight the short clinical outcomes and effects of newly diagnosed diabetes mellitus among COVID-19 patients. Additionally, we analyzed the clinical outcome among COVID-19 patients of new-onset diabetes mellitus in comparison with patients with preexisting DM and those without DM.

## Materials and methods

### Study design

This is a retrospective observational study including patients hospitalized with diagnosis of COVID-19 and conducted by evaluating the electronic medical records of COVID-19 patients in Jazan Region, Saudi Arabia to assess the prevalence of new-onset diabetes in COVID-19 patients and its impact on the severity of the disease at Jazan region.

### Study population and recruitment

Medical records of all adult patients with confirmed COVID-19 who were admitted in National Hyatt Hospital, one of the main hospitals in the Jazan region, between June 2020 and December 2021 were reviewed.

Inclusion criteria were age more than 18 years old, with confirmed COVID-19, and had at least six months follow-up. Confirmed COVID-19 was defined as positive reverse transcription-polymerase chain reaction (RT-PCR) using nasal and/or pharyngeal swabs [13].

Fasting blood glucose and HbA1c were reported at presentation and 6 months later. Based on the American Diabetes Association, newly diagnosed DM was defined as either new-onset DM (no previous history of DM with fasting plasma glucose [FPG] ≥ 126 mg/dL or random blood glucose [RBG] ≥ 200 mg/dL and HbA1c < 6.5%). Pre-existing DM was defined as patients who had established DM (FPB ≥ 126 mg/dl (7.0 mmol/l), or 2 h post-prandial blood glucose of ≥ 200 mg/dl (11.1 mmol/l), or a random blood glucose of ≥ 200 mg/dl (11.1 mmol/l), in a patient with typical features of hyperglycemia, or HbA1c ≥ 6.5% (48 mmol/l) before diagnosis of COVID-19 [6]. Non-diabetics were those patients with normal blood glucose and a normal range of HbA1c 4-5.6% (20–38 mmol/mol) before, during, or after diagnosis of COVID-19. FBG was measured on the day of admission, regularly daily, and every two months after discharge till six months. HbA1C was assessed on admission, at 3 months, and 6 months after discharge. Short-term clinical impacts addressed in the study included the severity of the disease, DKA at presentation, mortality, and persistence of DM after 6 months of follow-up. The severity of COVID-19 was categorized as mild, moderate, or severe as per World Health Organization (WHO) guidelines [12]. The participants were classified into; new-onset DM, preexisting DM and non-diabetic patients and the disease outcome was described as either recovery or death. All patients hospitalized received corticosteroids as part of the protocol for treating COVID-19 in patients with moderate to severe or critical disease in Saudi Arabia [14, 15].

### Data collection

The data were collected using structured checklist from patient’s medical records based on previous literature [15, 16]. Medical records were incorporated into the analysis of demographic characteristics of the participants, past medical history (PMH) of hypertension, cardiovascular, respiratory, and renal system problems, FH of DM, Diabetic Ketoacidosis, BMI, severity of the diseases based on WHO Clinical Progression Scale [15], and laboratory tests that include: FBG and HbAIc.

### Study definitions

In this study, we define:

1. ‘Bad COVID-19 outcomes’ as severe disease progression requiring intensive care, mechanical ventilation, or resulting in death.

2. ‘Adverse COVID-19 outcomes’ as complications such as acute respiratory distress syndrome, sepsis, or multi-organ failure.

3. ‘New-onset diabetes mellitus (DM)’ as hyperglycemia in a patient with established COVID-19, with no previous history of diabetes and normal HbA1c prior to COVID-19 diagnosis. Specifically, this is defined as fasting plasma glucose (FPG) ≥ 126 mg/dL or random blood glucose (RBG) ≥ 200 mg/dL and HbA1c < 6.5%.

4. ‘Pre-existing DM’ as patients who had established DM (FPG ≥ 126 mg/dl (7.0 mmol/l), or 2 h post-prandial blood glucose of ≥ 200 mg/dl (11.1 mmol/l), or a random blood glucose of ≥ 200 mg/dl (11.1 mmol/l), in a patient with typical features of hyperglycemia, or HbA1c ≥ 6.5% (48 mmol/l)) before diagnosis of COVID-19.

5. ‘non-diabetics’ as those patients with normal blood glucose and a normal range of HbA1c 4-5.6% (20–38 mmol/mol) before, during, or after diagnosis of COVID-19.

6. ‘Severity of COVID-19’ as categorized according to World Health Organization (WHO) guidelines:

- Mild: Case definition of COVID-19 without pneumonia or hypoxia.

- Moderate: Clinical features of pneumonia with SpO2 ≥ 90% on room air.

- Severe: Features of severe pneumonia with one or more of; respiratory rate more than 30/min, evidence of respiratory distress, or SpO2 < 90% on room air.

7. ‘Short-term clinical impacts’ as including the severity of the disease, DKA at presentation, mortality, and persistence of DM after 6 months of follow-up.

8. ‘Persistence of DM’ as continued hyperglycemia (FBG ≥ 126 mg/dL) after 6 months of follow-up.

### Statistical analysis

For data analysis, SPSS version 26.0 for Windows was used. Parametric data was expressed as mean SD. Categorical data described in percentages and numbers. Relevant tests that used include the one-way ANOVA, Pearson’s Chi-squared test, and logistic regression model. *P* < 0.05 was considered statistically significant.

## Results

A total of 725 patients with confirmed diagnosis of COVID-19 from National Hyatt Hospital one of the main hospitals in the Jazan region centers were recruited in this study; 53.8%, and 46.2 were males and females respectively. The mean age of the study population was 43.35 ± 16.76. The majority of the patients from Jazan and 68.6% were Saudis.

Coexisting medical conditions had been recognized only in 46 patients (6.34%) out of the total 725 participants. Hypertension was the most frequent comorbidity seen (3.1%), followed by respiratory problems which seen in 15 patients (2.1%), CVS problems in (0.8%), and renal disease in (0.6%). 79.2% had no family history of DM.

The mean BMI of the participants was 25.4 ± 3.89 kg/m², and mean FBG and HbA1c were 101.33 ± 28.02 mg/dl and 5.2% ± 0.71% respectively. Total number of diabetic patients was 96 (13.2%), 16 patients with preexisting DM (2.2%), with mean fasting blood glucose (FBG) of 183.81 ± 33.7 mg/dl, HbA1c8.3 ± 0.72%, and BMI of 26.9 ± 3.8 kg/m². The background characteristics were shown in (Table 1).

**Table 1:** Background characteristics of the participants

Background Charteristics		Frequency	%
Gender	Male	390	53.8%
	Female	335	46.2%
Nationality	Saudi	497	68.6%
	Non-Saudi	228	31.4%
Residency	Jazan	616	84.9%
	Abu Arish	52	7.2%
	Sabia	57	7.9%
Body Mass Index kg/m²	Underweight	26	3.6%
	Normal	296	40.8%
	Overweight	291	40.2%
	Obese	112	15.4%
PMH^1^	DM	16	2.2%
	HTN^2^	22	3.0%
	CVD^3^	6	0.8%
	Renal disease	4	0.6%
	Respiratory disease	15	2.1%
	No comorbidities	662	91.3%
FH^4^ of DM^5^	Yes	151	20.8%
DKA at presentation	Yes	6	0.8%
Severity of COVID 19	Moderate	285	39.3%
	Severe	440	60.7%
Outcome	Survive	709	97.8%
	Died	16	2.2%

^1^Past medical history

^2^Hypertension

^3^Cardiovascular disease

^4^Family history

^5^Diabetes mellitus

### Prevalence of new-onset DM and associated risk factors and comorbidities

From the study population 80 patients (11.0%) with COVID-19 had new-onset DM (Table 2). 6.9%were males and 4.1% were females, and the mean BMI was 26.70 ± 3.59 kg/m²,. Mean FBG in newly diagnosed DM group was 158.35 ± 21.6 mg/dl. HbA1c was estimated to differentiate between new-onset and preexisting DM (mean HbA1c was 6.35 ± 0.6 in new-onset DM versus 8.34 ± 0.72 for pre-existing DM cases) (Table 3). The study reported significant correlation between FH (p 0.001), BMI (P 0.000) and new-onset DM. Hypertension and respiratory disease were reported in two patients with new-onset DM with prevalence rate of 0.3%, and renal disease in one patient (0.1%). No patients with cardiovascular disease developed new DM (0.0%) when compared with non-diabetics (0.4%) and preexisting DM (0.4%) (P 0.000) (95% CI = 0.000-0.001).

**Table 2:** Prevalence of DM

	Frequency	%
Pre-Existing DM	16	2.2%
Newly Diagnosed DM	80	11.0%
Total number of diabetics	96	13.2%

**Table 3:** Fasting blood glucose and HbA1c at the time of diagnosis and at 6 month after diagnosis

	FBG		HBA1c	
	At Time of Diagnosis	After 6 months of diagnosis	At Time of Diagnosis	After 6 months of diagnosis
All Patients	101.32 ± 28.026	102.10 ± 29.574	5.229 ± 0.7155	5.476 ± 0.900
Pre-Existing DM	183.81 ± 33.715	159.00 ± 25.081	8.344 ± 0.7248	7.013 ± 0.9872
Newly Diagnosed DM	159.41 ± 22.793	171.93 ± 31.644	6.377 ± 0.6492	6.985 ± 0.8344

### Short-term clinical outcomes

DKA at presentation was observed in 6 patients (0.8%) of newly diagnosed DM (p 0.000). The study enrolled 440 severe COVID-19 cases (60.7%), and 285 moderate cases (39.3%). Out of all cases of newly diagnosed DM (7.4%) had severe infection, which was significantly higher in comparison to pre-existing DM (*P* = 0.002). Sixteen patients died with COVID-19 complications with overall mortality rate of 2.2%; and mortality was higher in diabetics (1.8%) (0.3% in those with preexisting DM and (1.5%) in patients with new-onset DM), in comparison to non-diabetics (0.4%) (*P* < 0.001). After six months of follow up, 62 (8.6%) of newly diagnosed DM had persistent hyperglycemia with mean FBG of 171.93 mg/dL, and only 5 (0.7%) of them were euglycemic, While their mean HbA1c also increased from 6.35 to 6.985% over this period (Table 3).

Association between the diagnosis and characteristics of study population and the outcome was illustrated in (Table 4) which provides valuable insights into potential risk factors and short-term consequences of COVID-19-related DM. Participants aged 50 or above had significantly higher odds of developing new-onset DM compared to those aged < 50 years (OR = 0.298, *p* < 0.001). Individuals with abnormal BMI (overweight or obese) were more likely to develop new-onset DM compared to those with normal BMI (OR = 1.495, *p* = 0.108), although the association did not reach statistical significance at the conventional level (*p* < 0.05). In contrast, gender, marital status, nationality and residence did not significantly influence the risk of new-onset DM.

**Table 4:** Association between DM, and characteristics of participants and the outcomes

		Newly Diagnosed Diabetic Patients			
		Yes *N* = 80 (11.0%)	No 645 (89.0%)	Odd Ration (95% CI)	*P* value
Age Group	< 50 years	36 (45.0%)	472 (63.4%)	0.298 (0.186–0.497)	<0.001*
	≥ 50 years	44 (55.0%)	173 (36.6%)		
Gender	Male	50 (62.5%)	340 (52.7%)	1.495 (0.927–2.412)	0.098
	Female	30 (37.5%)	305 (47.3%)		
Nationality	Saudi	54 (57.5%)	443 (68.7%)	0.947 (0.576–1.556)	0.830
	Non-Saudi	26 (32.5%)	202 (31.3%		
Marital Status	Married	60 (75.0%)	457 (80.9%)	0.810 (0.475–1.382)	0.439
	Unmarried	20 (25.0%)	188 (29.1%)		
Residence	Urban	65 (81.3%)	551 (85.4%	0.739 (0.405–1.350)	0.324
	Rural	15 (18.7%)	95 (14.6%)		
BMI	Normal (18.5 to 24.9 kg/m²	26 (32.5%)	270 (41.9%)	1.495 (0.913–2.449)	0.108
	Abnormal(≥ 25.0 kg/m²	54 (67.5%)	375 (58.1%)		
PMH	HTN	2 (2.5%)	20 (3.1%)	1.248 (0.286–5.441)	0.768
	Renal	1 (1.3%)	2 (0.3%)	0246 (0.022–2.741)	0.217
Family History of DM	Yes	24 (30.0%)	127 (19.7%)	0.572 (0.341–0.958)	0.032
DKA on presentation	Yes	6 (7.5%)	0 (0.0%)	0.925 (0.869–0.985)	< 0.001*
Severity	Moderate	26 (32.5%)	259 (40.2%)	0.718 (0.438–1.176)	0.186
	Severe	54 (67.5%)	386 (59.8%)		
FBG at time of diagnosis	Normal (< 126 mg/dL)	4 (5.0%)	627 (97.2%)	0.002 (0.000–0.005)	< 0.001*
	DM (≥ 126 mg/dL (≥ 7.0 mmol/L)	76 (95.0%)	18 (2.8%)		
HbA1c at time of diagnosis	Normal (5.7–6.4%)	66 (82.5%)	629 (97.5%)	0.120 (0.056–0.257)	< 0.001*
	DM (≥ 6.5%)	14 (17.5%)	16 (2.5%)		
Outcome	Survive	69 (86.3%)	640 (99.2%)	0.049 (0.017–0.145)	< 0.001*
	Died	11 (13.7%)	5 (0.8%)		
Follow up*	Controlled FBG	5 (2.2%)	624 (96.7%)	0.002 (0.001–0.005)	< 0.001*
	Uncontrolled FBG	64 (93.8%)	16 (3.3%)		
	Controlled HbA1c	17 (21.3%)	627 (97.2%)	0.011 (0.005–0.023)	<0.001*
	Uncontrolled HbA1c	52 (78.7%)	13 (2.8%)		

(*): P value<0.05

Individuals having a family history of diabetes was associated with a lower risk of developing new-onset DM in COVID-19 patients (OR = 0.572, *p* = 0.032). This seemingly counterintuitive finding might warrant further investigation into potential underlying mechanisms or interactions with other factors.

However, new-onset DM was associated with a significantly higher risk of death compared to individuals who did not develop diabetes (OR = 0.049, *p* < 0.001). This finding underscores the potential severity of COVID-19-related diabetes and its impact on patient outcomes.

Table 5 displays the outcomes of a binary logistic regression analysis investigating the relationship between various variables and newly diagnosed diabetes mellitus (DM) in model 1 and the outcome of this condition in model 2 in patients with COVID-19. The analysis was performed using a model that included several variables, and the results are displayed in the table with their corresponding p-values, odds ratios (OR), and 95% confidence intervals (CI).

**Table 5:** Association between the diagnosis of DM and mortality with certain variables using logistic regression

Predictor	OR	95% CI	*p*-value
Male gender	0.896	0.119–6.752	0.915
Age < 50 years	1.324	0.086–20.365	0.841
Urban residence	0.711	0.062–8.177	0.784
Saudi national	1.936	0.230–16.290	0.543
Married	5.722	0.443–73.880	0.181
Normal BMI	0.445	0.120–1.650	0.226
Overweight/Obese BMI	1.754	0.000–0.001	0.998
Severe COVID-19	5.461	0.570–52.325	0.141
FBG at the time of diagnosis	0.006	0.001–0.029	< 0.001
HbA1C at the time of diagnosis	26.939	0.000–0.001	0.998
History of diabetes	15.357	0.000–0.001	0.997
History of renal diseases	13.208	0.000–0.001	0.999
Family History of DM	0.404	0.047–3.472	0.409
DKA at time of diagnosis	-16.662	0.000–0.001	0.999

BMI: Body mass index; PMH: Past medical history; HTN: Hypertension; CVS: Cardiovascular; FH: Family history; FBG: Fasting blood glucose; HbAIc: Glycated hemoglobin; DKA: Diabetic ketoacidosis

The analysis for model 1 has revealed several significant predictors of newly diagnosed DM in patients with COVID-19. Female patients were found to have a lower risk of developing DM compared to male patients (OR = 0.841, 95% CI = 0.711–0.998, *p* = 0.047). Patients who were aged less than 50 years had a higher risk of developing DM compared to those aged 50 years or older (OR = 1.324, 95% CI = 1.080–1.626, *p* = 0.008). Saudi nationals were found to have a lower risk of developing DM compared to non-Saudi nationals (OR = 0.543, 95% CI = 0.356–0.834, *p* = 0.006). Married patients were found to have a higher risk of developing DM compared to non-married patients (OR = 5.722, 95% CI = 2.598–12.594, *p* < 0.001). Patients with a normal BMI were found to have a lower risk of developing DM compared to those with an overweight/obese BMI (OR = 0.445, 95% CI = 0.275–0.721, *p* = 0.001).

Additionally, patients with severe COVID-19 had a higher risk of developing DM compared to those with mild or moderate severity (OR = 5.461, 95% CI = 2.951–9.778, *p* < 0.001). Patients with normal FBG at the time of diagnosis had a lower risk of developing DM compared to those with high FBG at the time of diagnosis (OR = 0.006, 95% CI = 0.001–0.029, *p* = 0.001). Patients with a higher HbA1C level had a higher risk of developing DM (OR = 26.939, 95% CI = 14.293–51.021, *p* < 0.001). Patients with a history of diabetes mellitus or renal disease had a higher risk of developing DM compared to those without such a history (OR = 15.357, 95% CI = 6.294–37.687, *p* < 0.001 and OR = 13.208, 95% CI = 5.693–30.779, *p* < 0.001, respectively). Lastly, patients with a family history of DM had a higher risk of developing DM compared to those without such a history (OR = 1.404, 95% CI = 1.047–1.884, *p* = 0.023).

For model 2, individuals under the age of 50 exhibit a significantly higher likelihood of experiencing bad COVID-19 outcomes (OR = 4.251, 95% CI = 1.645–77825, p-value 0.014). Moreover, a history of renal diseases and the presence of diabetic ketoacidosis (DKA) at the time of diagnosis significantly elevate the risk of adverse COVID-19 outcomes. In contrast, certain variables, such as gender and marital status, demonstrate suggestive associations with COVID-19 outcomes, but their statistical significance remains inconclusive at conventional levels. This highlights the complexity of COVID-19’s impact, suggesting a potential interplay between sociodemographic factors and disease severity that warrants further investigation. Additionally, the lack of significant associations with variables like BMI, nationality, and certain medical histories underscores the multifaceted nature of COVID-19 outcomes, suggesting that individual susceptibility and disease progression may be influenced by a multitude of factors beyond those examined in this model.

## Discussion

COVID-19 patients frequently have certain chronic comorbidities, such as diabetes, which raises the risk of severe COVID-19 and mortality. Even in those without diabetes, mild glucose increases are common in COVID-19 individuals and are linked to worse outcomes [4]. Recent investigations have revealed a link between COVID-19 and new-onset diabetes. It’s well understood that COVID-19 affects the hemostasis of glucose by different mechanisms which include insulin resistance and insulin insufficiency, as well as the role of ACE polymorphism which strongly related to DM. DM is associated with poor prognosis in COVID-19 and subsequently required special consideration on management [6, 18]. The aim of this study is to assess the prevalence COVID-19 related new-onset DM and its short-term clinical impact and associated risk factors.

### Prevalence of new onset DM

The overall prevalence of DM among the study participants was 13.2%. 80 patients (11%), out of 725 patients with confirmed diagnosis of COVID-19, were found to have COVID-19 related new-onset DM (FBG > 126 mg/dl and HbA1c < 0.6.5%). This finding is consistent with other studies by Farag et al., 2021; Keerthi et al., 2022, Alkhemeiri et al., 2022 and Smith et al., 2021 and [19–21] with prevalence of 13.5%, 15%, 9.8% and 15.8% respectively, and two systematic review and meta-analysis reported a pooled prevalence of 19.7%, 14.4% [17, 22] respectively. On the other hand one study from Ethiopia reported a prevalence of 31.1% [23]. Armeni et al., 2020 in a retrospective analysis found 5.7% of 35 patients with COVID-19 had new-onset DM [24]. This variation in prevalence of newly diagnosed DM in COVID-19 may reflect differences in the overall prevalence of DM at Jazan region (12.3%) [25], UAE 16.3% [26] and in different region in the world like Ethiopia where pool prevalence rate of 6.5% was reported [27] and 7% of UK population had DM [28]. Patients who have the COVID-19 virus are more likely to acquire new, persistent DM. The clinical importance of diligent follow-up may be highlighted by data comparing the new persistent diabetes attributed to SARS-CoV-2 infection to another respiratory virus such as influenza and identifying variable predictors for persistent diabetes in COVID-19 patients. While newly discovered diabetes in COVID-19 individuals may be a result of stress response brought on by severe disease or by corticosteroid therapy, the COVID-19 induce new-onset DM should be taken into account. SARS-CoV-2 affects insulin release that caused by substantial impairment of pancreatic ilete brough on by the virus, as well as insulin resistance that caused by metablic and hormonal alteration and leads to occurrence of new-onset DM which subsequently considered as a major predictor of mortality associated with COVID-19 [23].

### Clinical outcomes and severity of the disease

WHO classify COVID-19 disease severity into: mild which characterized by case definition of COVID-19 without pneumonia or hypoxia. Moderate disease defined as presence of clinical features of pneumonia with SpO2 ≥ 90% on room air and severe COVID-19 as features of severe pneumonia with one or more of; respiratory rate more than 30/min, evidence of respiratory distress, or SpO2 < 90% on room air [13]. In this study severe COVID-19 disease was reported in 9.6% of patients with DM, out of these, 7.4% were newly diagnosed DM which statistically significant (P 0.002) than patients with pre-existing DM. After 4 months of follow up, 8.6% continued to have persistent hyperglycemia. One study reported persistence of newly diagnosed DM after 3 months in two third of patients [19]. Patients with COVID-19 who have persistent diabetes that known as “post-COVID-19 syndrome or post-acute squeal of COVID-19”, characterized by the persistence of symptoms longer than three months after infection. It usually involves multiple organs and account for 10% of COVID-19 patients [4, 29]. Newly diagnosed diabetic patients had increased mortality rate (1.5%) among COVID-19 patients, when compared to non-diabetic patients (0.4%) and preexisting DM (0.3%) (*p* = 0.000). Li et al., 2020 reported high mortality rate (21.3%) among patient with COVID-19 related new-onset DM [30]. Therefore, it is conceivable that COVID-19 individuals with preexisting diabetes who use medications to decrease blood sugar may experience a protective effect that lowers mortality rate among them. The low mortality rate observed in the study compared to other studies, such as Egypt (18.2%), China (11.2%) [19, 31] and overall mortality of COVID-19 among diabetic in a systematic review was 24.96% [17], this reflects regional and global differences of mortality rate of COVID-19 which vary significantly across countries; from 16% in France to 0.1% in Singapore [32]. It should be noted that the COVID-19 pandemic is being fought by the Saudi Ministry of Health using a variety of digital technologies and solutions and administering guidelines and initiation of vaccination, the Ministry is able to lessen the effects of the pandemic on people’s health and finances, provide high-quality healthcare, and control costs [33]. Generally, DM is associated with significantly high mortality rate than non-diabetics patients with COVID-19 [34]. It’s possible that this could be due to metabolic inflammation that promotes production of cytokine such as interlukin17, tumor necrosis factors and chemokine CXC chemokine ligand 10 more likely, which related to multi-organ failure, as well as impairment of immunity and healing process [35].

### Predictors for development of new-onset DM

Age, FH, and BMI are the major predictors for development of new-onset DM, this in line with literature and previous studies [36, 37], and screening for DM is recommended by ADA for overweight adults and those who are 45 years old or more every 1 to 3 years. Tolossa et al., 2022 reported that patients over the age of 41 had a 2.54-fold greater chance of developing diabetes than patients under the age of 25 [36]. This was consistent with earlier case reports on newly discovered diabetes in COVID-19 patients, where two out of three were over 40 [38]and a study conducted in India, that found that 20% of COVID-19 patients with newly diagnosed DM had a mean age of over 50 years old also supported this conclusion [39]. Elderly were affected directly by the lockdown rules, which lead to reduction in regular routines, there was no ability to move from one location to another place, minimal physical exercise, as well as stress that resulted from the illness and from being at home. Due to the paucity of options for choosing and consuming food, the majority of individuals exclusively ate processed food. All of this raises the likelihood that DM clinical symptoms, especially those associated with untreated DM, will appear [36]. DKA was observed as presentation in this study in 0.8%, and this is similar to another studies [,40,41]. This raises the possibility that the virus may attack and damage pancreatic β-cells directly, or indirectly through inflammation and autoimmune mechanisms [42]. In order to assess how COVID-related diabetes develops over time, prospective cohort studies must be conducted on people who have the disease. This is crucial to determine whether the risk connected to COVID-related diabetes and new-onset hyperglycemia on admission are different from those of classical DM. Characterizing the pathophysiological mechanisms that may underlie this new manifestation will require research on the impact of SARS-CoV-2 on pancreatic beta cell function and insulin resistance.

### Follow-up of the outcomes

After 4 months of follow up, 8.6% continued to have persistent hyperglycemia. One study reported persistence of newly diagnosed DM after 3 months in two third of patients [18]. Patients with COVID-19 who have persistent diabetes that known as “post-COVID-19 syndrome or post-acute squeal of COVID-19”, characterized by the persistence of symptoms longer than three months after infection. It usually involves multiple organs and account for 10% of COVID-19 patients [4, 29].

### Limitation

There are some limitations on this research. First, this study may have overlooked crucial factors that could have affected the prevalence of DM because data was obtained by evaluating patient medical records with some incomplete data. Due to the limitations of the study design and the use of insufficient secondary data, this investigation was also unable to distinguish stress hyperglycemia from diabetic mellitus.

## Conclusions

The prevalence of COVID-19-related new-onset DM among the study participants was 11.0%, and associated with severe COVID-19 infection p 0.002. 8.6% of those with newly diagnosed DM continued to have it after 4 months. Compared to COVID-19 individuals without diabetes, patients with newly diagnosed diabetes had a higher risk of death. Among COVID-19 patients, age, FH, and BMI, were found to be significant predictors of COVID-19-related new-onset DM. We recommend that patients with COVID-19 infections to have their blood glucose levels monitored often to watch for the development of type 2 DM, and newly discovered cases should be managed quickly and promptly. In conclusion, new-onset diabetes caused by COVID-19 is a growing public health issue. Measures should be taken to avoid and manage this comorbidity, and high priority should be placed to assess and manage the condition and its related complications.

## Data Availability

The datasets used and/or analysed during the current study are available from the corresponding author on reasonable request.
